# Review of Professor C. N. Peirce’s Recent Conclusions in Regard to Pyorrhœa Alveolaris

**Published:** 1894-04

**Authors:** James Truman


### REVIEW OF PROFESSOR C. N. PEIRCE'S RECENT CONCLUSIONS IN REGARD TO PYORRHŒA ALVEOLARIS.[1]

BY JAMES TRUMAN, D.D.S.

So much has been written on the subject of pyorrhœa alveolaris without any remarkable results following, that I should prefer not to enter the list of disputants, and therefore comply with the request of Dr. Butler with some reluctance, for, in my judgment, it would be better to wait for exact observations than risk possibly crude opinions upon a subject of so much importance as this may prove to be.

The paper is, however, of such a radical nature that it invites discussion, and we are forced to meet it by, at least, past and present experience, or admit that the author's conclusions are correct.

The premise laid down by Professor Peirce is, that we have in

---

[1] Read before the Eighth District Dental Society, Buffalo, N. Y., February 26, 1894.

the past been working under a mistake, and that pyorrhœa alveolaris, instead of occurring as a single pathological condition, is in fact a dual presentation, and must be considered from separate stand-points, for he says, "From a careful study of the abnormal pericemental—or rather the alveolo-cemental—membrane, it appears to me that we must recognize two closely allied, but yet very different, pathological states; different, as I shall attempt to demonstrate, in their *etiology*, in their *clinical history*, in their *symptomatology*, and in their *susceptibility to treatment*. . . . In one form of pericementitis the origin of the calcic salt is the saliva, and in the other form, the blood. The former I shall designate as *ptyalogenic* calcic pericementitis, expressive of the idea that in its origin it is *local*, *peripheral*, and *salivary*. The latter I shall designate as *hæmatogenic* calcic pericementitis, expressive of the idea that in its origin it is *constitutional*, *central*, and *associated* with some modification of the normal composite of the blood-plasma."

As this view is so important, I propose to examine it, for not only does it constitute the basis of his argument, but is a wide departure from accepted conditions surrounding this disease.

It is a well-known pathological fact that calcic deposits will not be made, in the majority of cases, in tissues unless there be preexisting changes present indicating impaired function or absolute death of the part. This being recognized as true, what must be the condition of the apices of the roots of teeth to admit the deposit upon their surfaces? The pericementum must have been in a state of inflammation through an antecedent irritation. This may have been produced by any of the usual disturbances, such as undue pressure, loss of pulp, etc.

It is, therefore, not an impossibility that deposits may occur at these points after destruction of the pericementum; indeed, it must be regarded as a very probable result in gouty subjects.

Before accepting this statement, admitting for the sake of argument that it occurs, it may be well to ask what are the phenomena attending this disease, pyorrhœa alveolaris, as generally understood. There is first an irritation of the pericementum, followed by a gradual destruction of that organ, and synchronously that of the periosteum of the alveolar process. The gum tissue is not primarily involved. The inflammation engendered by the progress of the disease produces pus. Microscopic investigations have proved the existence of pathogenic bacteria. Deposits may or may not be present; generally, in my experience, they are absent. When present, they are, in my judgment, of secondary importance and cannot be considered as vital to the progress of the disease. These constitute the main presentations at the gingivæ. If this disease be present at the apex of the same root, the manifestations and results must be similar in all respects; pus must be the product of inflammation, and this will find an outlet. The final result is a fistula. Consequently, apical inflammation means eventually destruction of the adjoining tissue, or a condition known as alveolar abscess. If this be true, then the pathology of this disease is well understood and needs no new nomenclature to explain it.

It seems to me to be impossible, and contrary to known pathological laws, that a deposit can be tolerated on a weakened membrane, or one which will subsequently become devitalized by its presence, and the pulp still continue in full activity.

When the subject is viewed in the light of experience, it has not been my observation that any such condition exists at the apical surfaces of teeth except as a result of abscess, and then only in rare instances. Whether in these the calcic deposition will give in all cases the uric-acid reaction remains to be proved, and would require an analysis in every case to demonstrate it. Admitting that it could be demonstrated in every case, it by no means settles the question of the origin of pyorrhœa alveolaris, though we may be forced to admit it as one of the factors in its production.

The reasoning of Professor Peirce in regard to the channel by which uric acid arrives at the pericementum, and through the cementum and the dentine, is open to criticism. He says, "As the current of the lymph-stream is directed for the most part towards the cementum, through its borders or periphery into the lacunæ and canaliculi, and finally in the reverse direction, it is not difficult to see why the deposit should take place on the surface of the cementum, as well as in the meshes of the alveolo-cemental membrane. The constant deposition and pressure of these insoluble salts will act as irritants, engendering the well-known inflammatory states,—viz., congestion, exudation, impaired nutrition, tissue disorganization, and the formation of pus. These changes, taking place here as elsewhere, in the immediate vicinity of the irritation,—that is, on the cemental aspect of the membrane,—lead to its detachment from the cementum and the development of a pus-pocket."

When this quotation is analyzed, it represents the lymph-stream holding the urates in solution as passing into the lacunæ and canaliculi of the cementum without apparently affecting the tissues in transit. On their return, in reverse order, the stream gives up its calcific material, and the precipitation and crystallization result in irritation. As I have endeavored to show, there must be some loss of power or impairment of function for this deposition to take place; it is reasonable to infer that if the tissue retains full vitality, there would be no formation of calcic deposits. But Professor Peirce does not seem to regard this as an important pre-existing condition, though he does admit that there "might coexist some impairment of nutrition . . . or some faulty innervation." If this be not important, then it is difficult to understand why the deposit is not made before the transit through the pericementum. Why must the lymph-stream take such a circuitous route before it can effect the lesions described? If the urates be the irritants asserted, and impairment of nutrition is not absolutely essential to their deposition, no reason remains why the tooth should not go down to destruction as a whole, and not by degrees, as I believe to be universally the case.

In the discussion of the paper, and practically part of it, Professor Peirce makes the statement that "This disease [pyorrhœa] is not known to be developed, except in very rare cases, until after thirty or thirty-five years of age," and he reasons from this that, as the "uric-acid diathesis is not established until after thirty-five or forty years . . . we would not be likely to find any such condition in our young patients for whom we correct irregularities." This, by direct implication, means that there is a marked synchronism in these two pathological states. Unfortunately for this reasoning, pyorrhœa is *not* confined to thirty years of age, but is found very frequently in persons before the twentieth year; in fact, it is a question whether the age limit can be applied. It is true that after thirty-five the destruction is greater and the disease more deeply pronounced.

"In one form of pericementitis [pyorrhœa] the origin of the calcic salt is the saliva, and in the other the blood," is the rather positive assertion of Professor Peirce. At this stage of the inquiry it might be well, before ascribing a similar origin to both forms and both arising from calcic deposits, to establish the fact, if fact it be, that such deposits originate the lesion in any considerable number of cases. If by naming that originating from the saliva ptyalogenic calcic pericementitis, and that from the blood hæmatogenic calcic pericementitis, he means to assert that pyorrhœa alveolaris is invariably produced by calcic deposition, then equally as good observers must part company with him in his conclusions; for calcic accretions from the saliva, it is very safe to affirm, are not the cause of this disease. It is only necessary to refer, by way of illustration, to those teeth most affected with what he terms ptyalogenic deposits and mark the result. Examine the lingual surfaces of the inferior incisors and canines, and though ever so much coated with hard, black tartar, pyorrhœa does not result, and the same is equally true of the superior molars; indeed, as I understand the disease, it would be impossible for it to take place as long as salivary calculi were present.[1] If this be true of this character of calcic depositions, and it seems to me it cannot be controverted, then it remains equally true that the so-called hæmatogenic calculus can have no direct effect of a destructive character not possessed by the salivary calculus. Both alike destroy the soft tissues upon which they impinge, but neither, in my opinion, have any direct action in producing the disease in question.

The position held by Professor Peirce is that local treatment is practically unavailing, as it should be if his premises be correct; but inasmuch as pyorrhœa alveolaris has been and will continue to be overcome by local treatment, it follows that the uric-acid hypothesis does not cover all cases.

When it is understood that in dealing with this disease our diagnosis cannot be limited to a circumscribed area, but must have a wide consideration of related systemic conditions, I am forced to agree with the conclusions of the essayist, that lesions of the gingivæ may be indirectly aggravated by constitutional disturbances. It, however, remains to be proved that gouty subjects are, above all others, liable to this disease, or that to this disease can be accredited the origin of these peculiar pericemental inflammations. All disturbances of the normal equilibrium of the system will produce derangements of various organs, and a local irritation may be the progenitor of a series of new foci of inflammation.

A strict parity of reasoning would force the conclusion that if the hypothesis of Professor Peirce be the correct one, then we can equally ascribe the origin of pyorrhœa alveolaris to other disturbing elements. In scurvy and syphilis the pericementum is destructively affected, and under proper constitutional treatment will disappear, precisely as it is stated pyorrhœa will disappear under proper treatment for gout. Certain medicines act in the same way, and mercurial salivation is so well known as to need only passing reference.

---

[1] I do not wish to be understood by this statement that pyorrhœa does not occur on teeth upon which salivary calculus is deposited, but that it does not originate beneath the accretion.

The reasoning that would apply the origin to uric-acid deposits holds good equally for scorbutus, syphilis, and mercurial salivation.

It must be recognized that pathological conditions affecting the system will have a marked impression on the tissues investing the teeth, and in addition to this create changes in the secretions of the oral cavity, from the normal neutral to an increased acidity, thus locally intensifying the disease.

True pyorrhœa cannot, in my opinion, exist anywhere except upon the pericemental membrane investing the cervical portion of the tooth. It has its origin, if I understand it, in first irritation of that membrane, and may then progress, the factor of vitality having much to do with the rapidity of the destruction. The immediate and continuing cause of the inflammation is micro-organisms. As a rule deposits are not found in the pockets. Professor Peirce explains this by supposing that pus washes these out. If removed immediately, how can the parts be affected by their temporary presence?

Whenever differences arise in regard to the character and treatment of pyorrhœa the charge is almost universally made that those in opposition to any special ideas are incapable of diagnosticating the disease. It would be a sad commentary on the intelligence of the dental profession if this were true, but it is not true, for the reason that the very simplicity of the disease precludes such an inference. I am, therefore, forced to the conclusion that the marked differences of opinion cannot be attributed to a lack of knowledge of the etiology of this complaint, but rather to improper conceptions of treatment demanded.

The object of this review is not to give my own methods to meet these conditions, for this would be unnecessary, having been published elsewhere, but as teeth have been permanently cured, and that without any resort to systemic treatment for gout or other constitutional diseases, it seems reasonable to conclude that the origin of pyorrhœa cannot universally be looked for in derangements of the general system.

In conclusion, I briefly give the following summary of my views regarding the matter.

1. Pyorrhœa does not occur at the apices of roots as a distinct pathological condition.

2. Deposits may be found there, *but always subsequent to the death of the pulp and pericemental membrane.*

3. That uric acid may affect the membrane subsequent to the injury of that tissue, but is not directly the producing cause of lesions in that organ.

4. The action of syphilis, scurvy, certain medicines, and general constitutional disturbances have a similar effect to invite this pathological state.

5. Pyorrhœa alveolaris is, finally, a local disease, aggravated by systemic conditions, and to be treated upon a clear comprehension of its local and constitutional complications.

---

